# An Integrated Approach to the Conceptualisation and Measurement of Social Cohesion

**DOI:** 10.1007/s11205-023-03110-z

**Published:** 2023-05-25

**Authors:** Bujar Aruqaj

**Affiliations:** Institut Für Soziologie, Arbeitsbereich Makrosoziologie, Freie Universität Berlin, Garystr. 55, Raum 314, 14195 Berlin, Germany

**Keywords:** Social cohesion, Social integration, Social trust, Social inequalities, Latent conflict, Europe, Social cohesion index, Subjective well-being, Regional cohesion

## Abstract

The core sociological subject of ‘social cohesion’ (hereafter SC) has re-emerged as a key concept in the social sciences. On the one hand, SC is thought to be influenced by a society’s degree of inequalities and the quality of its welfare state. On the other hand, SC is thought to be instrumental in its own right to other factors such as economic growth, institutional quality, and individual well-being. In recent years, a few attempts have been made to measure SC empirically. Many current indices have not been sufficiently theoretically substantiated, and do not consider the importance of different ‘social levels’ when explaining and measuring SC as both cause and effect of other correlates. Very often, SC is simply defined as a ‘social quality’ or a quality of a collective. As a result, measures are often aggregate macro-indices leading to a loss of the information base of any social ‘units’ below the macro-societal-level. Contributing to this important methodological debate, this paper provides a conceptual reformulation of SC. Hence, when assessing SC based on a multi-dimensional index, it is insightful and feasible to evaluate both its internal variation as well as its holistic validity. In fact, it is proposed that these two aspects of measurement stand in direct relationship to one-another. The paper starts out with a discussion of SC as a ‘social fact’ in the Durkheimian sense. In addition, three bridging propositions on the measurement of SC are advanced: (a) SC as outcome or consequence at the level of individual attitudes and orientations (‘micro’); (b) SC as degree of dissimilarity and presence of latent conflict within a society at the level of salient social categories (‘meso’), and (c) SC as predictor, social determinant and hence antecedent at the societal-level (‘macro’). Using all rounds of the European Social Survey with a very large sample size, the advantages of this approach are illustrated by singling-out the important link between socio-economic inequalities, social cohesion and individual subjective well-being in a path of action.

## Introduction

It would be fair to say that the question of social cohesion (in other words—‘what keeps a society together?’) is as old as the discipline of sociology itself, and has preoccupied, in one way or another, many of the most influential names within the field (Durkheim, 2013; Gellner 1983; Giddens, 1986; Lockwood, 1999; Parsons, 2005). From the writings of Durkheim and onwards, the concept has frequently arisen during times of major socio-economic transformation (i.e., secularisation, individualisation). It is therefore not surprising that in the 1990s the concept became an important policy concern, as a “reaction to certain strategies of accommodation to conditions of international economic competition and restructuring that occurred in the 1980s and 1990s” (Jenson, 1998: 5). SC has again become a concept at the core of the debate around globalisation and the fate of modern societies. Initially, a growing number of European scholars became concerned with SC in the aftermath of the so-called ‘refugee crisis’, a development linked with popular fears of the potentially harmful impact of diversification on SC. While the literature remains divided on the subject, a significant number of studies so far have challenged Putnam’s (2007) findings that heterogeneity has a negative effect on SC per se (see Meer & Tolsma, 2014). The link between heterogeneity and cohesion is said to be highly mediated by economic deprivation (Letki, 2008: 121) as well as inter-group contact (Green & Wong, 2009: 228; Marschall & Stolle, 2004: 130). In the wake of the turmoil of great global migration movements, we have seen the establishment of major governmental and non-governmental initiatives for the further research of SC, particularly in Germany (Arant et al., 2016; BMBF, 2017; BUA, 2020). The increased focus the academic community has given to SC was made clear once again in March 2020, when the novel coronavirus pandemic prompted fears about the consequences of heavily-reduced social interaction, due to ‘lockdowns’ and ‘social distancing’, and its impact on SC in affected societies.

Despite the attention given to the topic, there is a longstanding agreement in the literature that SC is a rather nebulous and ill-defined concept, one used in a wide variety of contexts (Chan et al., 2006: 274; Schiefer & van der Noll, 2017: 583). However, although this is generally accepted to be one of the main problems with the concept, few contributions focus on addressing it more specifically. Although SC is at times discussed in terms of different ‘levels’, the integrative interdependence of these levels in the context of SC is rarely addressed conceptually, and even more rarely operationalised empirically. Because there exists a rather broad consensus view of SC as a ‘holistic’ quality, many empirical studies have measured SC solely at the national-level (Berger-Schmidt, 2000; Delhey et al., 2018; Foa, 2011). Yet, this paper shows that we can define SC as a quality of societies, without necessarily being restricted to measuring it in a solely “methodologically nationalist” way (Wimmer & Schiller, 2002). The very meaning of societal cohesion presumes that we should be interested—also and particularly—in the internal composition of societies, hence their ‘cohesiveness’, and not only in comparisons between societies. The internal orientations within a society, in this view, might significantly explain the performance of societies when comparing them to one another. The value of this critique is further reaffirmed by the fact that many contemporary studies on SC have focused predominantly on between-country comparisons (Dickes et al., 2009; Midtbøen, 2015; Tokman, 2007) and the classification of countries in various ‘regimes of cohesion’ (Dickes & Valentova, 2013: 842; Dragolov et al., 2016: 51; Green & Janmaat, 2011: 118). Yet, assessing the quality of social relations and bonds *within* a society, among members of that society and salient social categorisations, seems just as essential to understanding SC. This information is in most cases entirely lost when SC is operationalised as a single value representing a whole society. The paper shows that in the case of a construct such as SC, different levels of analysis provide distinct forms of data, that function as *bridging* information on this phenomenon, rather than throwing up a “wall of non-inference” between them (Welzel & Inglehart, 2016: 1072).

Additionally, current empirical studies have considered macro-societal cohesion as both an *outcome* (consequence) and *predictor* (antecedent) of other events and processes, without further clarification (Dragolov et al., 2016: 59). Thus, in the broader literature generally levels of inequality, modernisation and development are all thought to be substantial predictors of SC, mostly measured as aggregate attitudes of trust (Bécares et al., 2011: 2779; Green et al., 2003: 543; Vergolini, 2011: 207). At the same time, SC is thought to be a “good in its own right” and to affect other desirable outcomes, most notably individual human well-being (Delhey & Dragolov, 2016: 165; Klein, 2013: 897) and economic output/productivity (Easterly et al., 2006: 103). This presumed ‘duality’ of SC as both cause and effect of other correlates is solely analysed at the societal-level, mostly through methods such as correlations, cross-country regressions and cluster analyses. The paper provides theoretical and empirical insights that are needed if we want to better explain how the cohesion of societies is embedded and affected by a broader socio-economic context, as well as how the cohesion of societies affects in turn other desirable outcomes. This paper offers a conceptual and operational framework for a distinctive measurement of SC as both outcome and predictor of other parameters. The measure allows us to create a much more detailed profile of SC within and between societies. Initially, SC is conceptualised as a “social fact” (Durkheim, [1895] 1982), to provide a more principal definition of the term, which is often used in scientific and political debates in a very unclear manner. Furthermore, an ‘integrated index’ of SC (SCI), which considers not only different dimensions, but also different social units as viable to the measurement of SC is operationalised, and its advantages are discussed. The strength of this conceptually-derived measure is illustrated in the results section, by progressively applying it to the individual (micro), regional (meso) and societal (macro) levels.

## Conceptualising SC as Social Fact

The totality of beliefs and sentiments common to the average members of a society forms a determinate system with a life of its own. It can be termed the collective or common consciousness […] it is something totally different from the consciousness of individuals, although it is only realized in individuals. It is the psychological type of society, one which has its properties, conditions for existence and mode of development, just as individual types do, but in a different fashion (Durkheim, [1893] 2013, 39).

Although in current studies of SC the works of Emile Durkheim are often only cited in passing, his insights into social integration and solidarity are indeed indispensable to the analysis of SC. Durkheim defines SC as a characteristic of societies related to the interdependence of individuals in that society. In a society exhibiting “mechanical solidarity”, its cohesion derives from the cultural homogeneity of individuals. In contrast, SC in advanced societies exhibiting “organic solidarity” is based upon the modern interdependence of diverse individuals on each other. All contemporary European societies can be thought to be defined by this type of organic solidarity. The question is only to what degree? In his work *Suicide*, Durkheim ([1879] 2005, 325) defines social cohesion as (a) the presence of strong social bonds in society, and (b) the absence of latent conflict, such as that based on wealth, ethnicity, race, and gender (Fonseca et al., 2019: 246). A further conceptual and analytical takeaway from Durkheim’s *The Rules of Sociological Methods* ([1895] 1982) and *The Division of Labor* ([1893] 2013), is that SC is a “social fact”, as can be inferred from the quotation above. Essentially, a social fact is a “social phenomenon that has a coercive effect upon the individual. Thus, although social facts may originally be the product of human action, they have developed an autonomy from their human authors, and now confront humans as something external to them” (Edgar & Sedgwick, 2007: 215). The association of individual human beings creates a social reality of a new kind (i.e., ‘sui generis’), and it is only in the facts of that association that the explanation for this new reality can be found (Durkheim, [1895] 1982, 23). Hence, it is truly only in this collective association that we can define SC, albeit it is not the only way in which we can measure it, as shall be argued here.

In sociology, the individualism–holism debate has been much discussed throughout the modern history of the discipline. While Durkheim advocated a holistic perspective, Max Weber theorised about an individualistic approach (Dahlback, 1998: 237). Weber ([1922] 1978, 71) wrote of “*Vergemeinschaftung*” (community formation) and “*Vergesellschaftung*” (society formation). However, contrary to Durkheim, Weber did not see these phenomena as rooted in social facts, but instead in the “affectual or traditional feelings of individuals in the case of community—and rational agreements by mutual consent (e.g. a commercial contract)—in the case of society formation” (Green & Janmaat, 2011: 21). According to Durkheim, however, individuals in society are not bound by any sort of rational contract, but by common norms. This antinomy is still relevant today, especially to the discussions on the measurement of SC. In particular, two crucial questions remain to be addressed: (a) Does SC refer to an “aggregate” of separate individuals, whose actions are not directly related to each other, at least not in a way that can be taken as relevant for the analysis?; (b) Can SC be entirely measured by looking at the societal (i.e. holistic) level alone, as is currently a common practise?

In a more recent contribution, Welzel and Inglehart (2016) made a distinction when measuring a multidimensional construct’s “internal convergence”—that is, the degree to which the items in a construct correlate at the individual-level—and a construct’s “external linkage”, or the degree to which convergence patterns at the aggregate (i.e., macro-level) “map in corresponding fashion on the construct’s supposed antecedents, outcomes, or correlates” (ibid: 1069). The conception here posits that it is possible and desirable when creating such indicators, to be able to preserve and assess both the internal convergence, as well as the external linkage of an indicator such as social cohesion. In agreement with Welzel and Inglehart, this conception posits that “convergence patterns at the aggregate-level exist in their own right”, a claim that resonates strongly with Durkheim’s ideas about social facts. However, this conception will take a more critical stance on Welzel and Inglehart’s other conclusion, that a “construct’s measurement features at the individual level provide *no information whatsoever* about the same construct’s validity at the country level” (ibid: 1070).

Here, a viable solution where we can consider both individualistic and holistic approaches as relevant to the explanation, and importantly the practical measurement of SC is advanced. The core propositions of this approach are that SC can be measured at multiple social levels, albeit as an integrated whole. First, SC should be measured at the level of individual orientations as outcome, consequence or manifestation of other factors. Here, we can ask for instance if the degree of inequities in a society affects orientations conducive to SC at the *individual-level* (i.e. influences the individuals’ situation and attitudes). Secondly, the ‘dissimilarity’ of SC orientations at the ‘group-level’ provides an important indication of the quality of social relations between different groups within a society and hence its cohesiveness. Here, we can ask if major within-country social categorisations (e.g. socio-economic classes) differ significantly in their orientations of cohesion. Finally, when we aggregate these orientations to the level of societies, we do not only obtain an indicator of the overall degree of SC in a society, but also SC as a social fact and hence a social determinant (i.e., antecedent) in its own right. Here we can ask, for instance, whether the societal degree of SC has an impact on individual well-being, human capabilities (Lanzi, 2011), sense of security and so on. Thus, SC can be thought of as being more directly affected on the level of individual orientations, while at the same time having an effect of its own when established at the collective-level.

Taking Durkheim’s social facts seriously, SC is a collective phenomenon, but the characteristics of solidarity (both mechanical and organic) are only observable in individuals. Thus, it can be claimed that the *concept* and the *measurement* ‘level’ essentially differ in the case of SC: the concept applies only to collectives, but the measurement is essentially done at the individual-level. Current country-indices of SC are usually obtained by averaging such individual attitudes. They are often collected at the individual-level, without being applicable to the individual-level. However, societal (i.e., macro) SC, although a distinct reality, is essentially a ‘product’ of the aggregation of these individual attitudes, or what I have called here individual ‘orientations’, to denote their directionality. The actions of these individuals are not directly related to each other, as with more interpersonal interactions and networks (e.g., social capital). In the case of SC, we are dealing with broadly generalised social bonds among fellow members of a society that enable its basic social togetherness. However, they are indirectly related to each other in the sense that members and salient social categorisations need to *share* to some degree these attitudes conducive to SC.

A key distinction needs to be drawn here between such orientations indicating more general social bonds on the one hand, and more specific individual human values on the other (see Schwartz, 2012). According to Durkheim, social order is possible only because individuals of different background share common norms and values, which unite them together on a cooperative basis. In many definitions, social cohesion is about this “connectedness” of members which is manifested mostly through perceived trust, helpfulness (Lockwood, 1999: 69), solidarity (Durkheim, [1895] 2013: 345), and cooperation of fellow members in society and importantly also with the institutions in that society. Parsons’ view of “value generality” (1971: 307); Rawls’ “well-ordered society” (1995: 133); Easton’s termed “support for the political community” (1957: 391); and Habermas’ notion of “constitutional patriotism” (1994: 6), although emphasising different concerns, essentially all point in the same direction: in pluralistic and diversified societies, such as in contemporary European ones, a normative consensus does not equal a value consensus in the strict sense, but rather agreement on a limited set of more ‘civic’ overarching values despite individual value differences. This is particularly the case for European societies, undergoing the Europeanisation project. These societies have to cope with a plurality of different backgrounds, beliefs, and values if they want to maintain cohesion (Berger, 1998: 63). One thing is evident, there seems to be a general agreement among scholars that togetherness based solely on value homogeneity (e.g., ethnic or religious), as it might have existed once, is becoming impossible to be achieved (Bernard, 1999: 12; Jenson, 1998: 21), and may in any case not be socially desirable (Harell & Stolle, 2011: 16; Hooghe, 2011: 9). The question is: which loyalties and bonds would indicate the degree of SC in these societies nonetheless? For Parsons (1971: 307) “the more differentiated the system, the higher the level of generality at which the value-pattern must be ‘couched’ if it is to legitimate the more specified values of all of the differentiated parts of the social system.” Contemporary egalitarian values, social trust and cooperation with fellow members and with institutions, as well as values of acceptance and civic identification would indicate this sort of normative consensus in European societies today. Aside from these essential classical contributions, almost all influential contemporary studies on the topic of SC, have also recognized the importance of attitudes related to social and institutional trust as well as ‘respect for diversity’ to SC (Langer et al., 2017: 325; Larsen, 2013: 5; Schiefer & van der Noll, 2017: 594; Schmeets & te Riele, 2014: 794). However, the extent to which these orientations are mutually shared within a society is largely neglected in these studies, as simple aggregate country-scores cannot provide this sort of crucial information. The paper will define these important conceptual components of SC once again when discussing the indicators and constructing a measure of SC in the later section. Beforehand, I would like to presents the integrated model for the measurement of SC on three distinct social levels.

First, as SC is ‘manifested’ in individual attitudes, the question that emerges concerns what the factors that influence these attitudes more directly are. For Durkheim the division of labour would certainly have been an important factor to account for. We can extend this notion to include more contemporary concerns such as income inequalities (Pickett & Wilkinson, 2010: 107; Rothstein & Uslaner, 2005: 41), and the level of modernisation and development of societies (Dayton-Johnson, 2001: 7), all of which have been consistently found to have an impact on SC. Furthermore, we cannot exclude the possibility that individual attitudes conducive to SC may also be affected by more individual circumstances, such as being unemployed or discriminated against (Harell & Stolle, 2010: 16). Therefore, as an outcome of such parameters, it is both more plausible and effective to measure SC by looking precisely at what affects individual SC orientations of members at this level of manifestation. As these subjective orientations are undoubtedly conditioned by a broader and more materialist socio-economic context, the measurement of individual orientations of SC carries an importance on its own, as outcome.

Second, moving one step ‘upwards’, we have to consider what can be termed the ‘meso-level’, which refers to smaller defined populations within societies. Although the term ‘meso’ may not be entirely appropriate, in our context, it posits an analysis which specifically aims to reveal connections between cross-group and societal-level cohesion. At this ‘middle’ or ‘group level’, we can consider the orientations with regard to SC as aggregated across different social categorisations such as ethnic or religious groups, regions within a society, or social classes. If these important social categorisations reveal significantly dissimilar orientations, we can take this as a proxy of “latent conflict” (Bartos & Wehr, 2002) within a society. Thus, we can suspect that the larger the dissimilarity between defined groups, the bigger the ‘loss’ of SC suffered by the society as a whole.

Third, when aggregating attitudes at the national-level, we do not only obtain a comparative estimate as to which societies are cohesive. We also obtain *societal* SC as an important macro-level condition in the sense of a social fact that exerts a coercive force over individual members in that society. As such, societal- or macro-level SC can also be conceptualised as a ‘good in its own right’ or as an enabling “social opportunity structure” (Merton, 1938). It is only in the collective association that SC can be thought of as such a structure and as having an effect as an antecedent in its own.

**Fig. 1:**
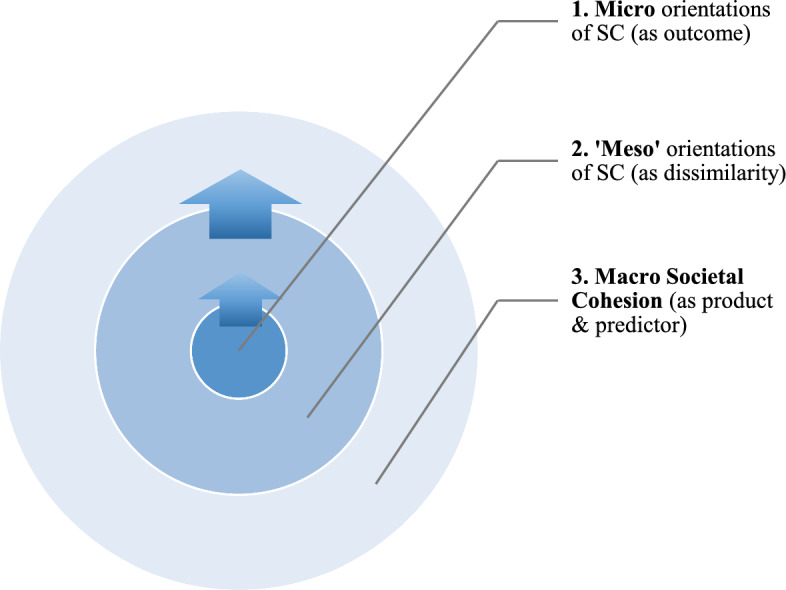
Conceptualising social cohesion

This conceptual reformulation departs significantly from many current approaches to SC which conceptualise and measure this concept exclusively as a macro quality, or at only one of these limited levels. Even when different social levels are analysed in the context of SC, the important link between them is not recognized. This conception provides an important additional tool for current studies for two main reasons: First, it prescribes to SC a logical directionality from the individual to the meso and then to the collective macro-level (Fig. 1). The macro-level in this case is the “referent of functional significance” as the ‘lower’ levels are of “functional contribution” to it (Parsons, 2005: 18), allowing us to understand how SC is jointly-established at the societal-level through members’ and groups’ cooperative orientations (i.e., social bonds). Second, it provides a viable solution in that it allows us to measure SC distinctly as both consequence and antecedent of other correlates, allowing us to deal with problems such as ‘endogeneity’ and ‘reverse causality’ more effectively. Thus, when considered as a consequence or outcome, it is advised to measure SC more directly at the level of individual attitudes or orientations. When considered as a predictor, it is advised to measure SC as an aggregate of collectives, as social fact. An important distinction which has been lacking so far in studies in this field. The dissimilarity of orientations of cohesion at the denoted ‘meso-level’ (however the ‘sub-societal’ units may be defined) within a society provides additional and essential insights into latent conflict, which erodes cohesion. If this is the case, such group cleavages would correlate with poor performance when compared to aggregate-societal cohesion. The approach acknowledges that while the measurement of SC means different things at different levels of analysis, it does so in an informative way that allows us to consider a more insightful profile of societies with regard to SC, within and between them. It is important to note that although the indicators I am about to select for this study may be context-specific to Europe, the conceptual reformulation I have proposed can plausibly be applied to other regions of the world (e.g., Africa, Latin America, Asia etc.) given appropriate data when considering what affects individual orientations to SC, how the within-country dissimilarities (based on e.g. class, religion etc.) may impede SC, and the possible effects that societal SC would have as an opportunity structure on its own.

## SCI: Measuring SC as Social Relationships

In this section, the construction of an integrated index of SC, the SCI, will be proposed and discussed. To be able to measure SC, we need to first define its constitutive components or concept properties. Although the main source of confusion seems to be a “proliferation of definitions of social cohesion that have proved difficult to combine or reconcile” (Friedkin, 2004: 209), for the majority of scholars it is at least very clear that SC is about ‘social relations’, ‘social bonds’, ‘cooperation’ and ‘interconnectedness’ (Alexandre et al., 2012: 6; Kalenkoski & Hamrick, 2014: 261; Wickham, 2002: 6). Furthermore, social cohesion is closely and traditionally interlinked with the concepts of social integration (Midtbøen, 2015: 12), solidarity (Friedkin, 2004: 418), and social trust in particular (Larsen, 2013: 5). But rather than being interpersonal and in more bounded groups, such as in the case of “social capital” (Coleman, 1988: 105), SC refers to a set of more generalised orientations that are ideally shared by members of a society. Generalised in this sense means a ‘blind’ application to all community members. Therefore, SC is also often described as the “social glue that binds all members in a society” (Schmeets, 2011: 128). This emphasis on understanding the factors that contribute to the ‘togetherness’ of society as a whole gives SC its conceptual distinctiveness. The sentiments and attitudes conducive of SC can therefore be best measured as a set of more bridging social relationships and bonds in a society as manifested in the orientations of individuals.

When considering both more classical (Durkheim, [1893] 2013; Lockwood, 1999) and more contemporary conceptions (Beauvais & Jenson, 2002: 9; Langer et al., 2017: 324; Lanzi, 2011: 1093), three types of social relationships seem to be essential to defining and empirically observing SC: (a) how members of a society relate to each other; (b) the relationship between different social groups within a society, and (c) how members relate to core state and social institutions. The quality of these social relationships is reflected in individual orientations such as generalised or social trust (relationship of all members), trust in institutions (relationship with institutions), and ‘acceptance’, ‘respect for diversity’ or what is called here ‘openness’ (relationships between different social groups). Social cohesion in differentiated societies can thus be broadly defined as the propensity of persons to *cooperate with each other as members of a society, across key cleavages, and with institutions.* When screening a large body of definitions pertaining to SC, most of them will fall into one or more of these categories. Yet, European definitions of SC tend to be characterised as more “institution-driven approaches”, while North American definitions tend to be characterised as more “societal-driven” (Hooghe, 2011: 7). A more accurate distinction would be that European definitions are more structurally-driven, by placing importance on inequalities and opportunities, while US approaches are more individually rather than societal driven. However, while many definitions focus on one of these relationships specifically, they negate the importance of the others. Thus, Larsen (2013: 10) recognises mostly the role of social trust and openness but does not mention the importance of institutional trust; Maxwell (1996: 13) recognises mainly the importance of inclusion and openness, and Berger-Schmidt (2000: 3) mostly that of social trust. Likewise, Schmeets (2011: 128), and Alexandre et al., (2012: 3) only cover social trust as an essential component of cohesion. The definition advanced here considers all three of these elements – that is, social trust, institutional trust, and openness as vital to the construction of SC. Certainly, Langer et al., (2017: 329), have recognised the importance of the relationship between social groups. However, in addition, we ought to measure these relationships more directly at different social levels, whenever the data permits. Consequently, SC forms a triad of the three latent dimensions: (a) social trust; (b) institutional trust, and (c) openness. From these elements, it is possible to construct a more precise and minimal definition of SC which can be used for research, analysis, and measurement of the concept. It follows that *the degree of social cohesion in a society depends on the extent to which members share attitudes of trust in each other and in institutions and the extent to which they accept each other’s differences.*

The use of a composite indicator in the case of SC is advisable given that this construct is composed of multiple dimensions. However, to be able to operationalise such an integrated SCI, a couple of key data criteria need to be met. First, in order to permit for multi-level assessment, the selected indicators need to be collected at the individual-level and need to derive all from a single dataset. They cannot be merged using different datasets with non-identical items, as is often the case, since this would lead to a loss of the information-base at the individual-level. The same items have to be also interchangeable across all rounds of a survey if the index is to also allow for longitudinal analysis. In this way, we can preserve a data continuity between social levels as well as across time. Secondly, as set out in the conceptual framework, the items need to reflect more generalised individual attitudes vis-à-vis other members of society, social groups and the institutions as thoroughly as possible. To the extent that surveys include markers for different social categorisations, the index can additionally allow for a more insightful observation of SC at the ‘meso-level’.

**Fig. 2:**
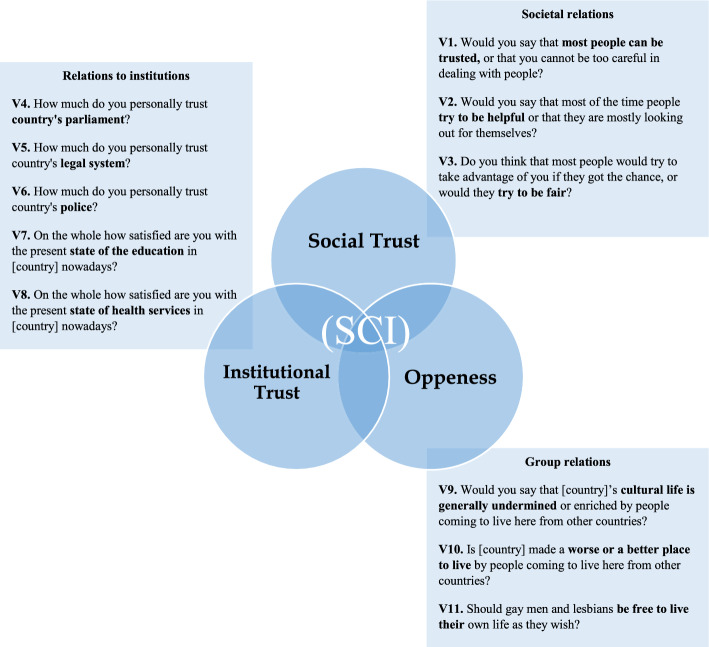
Social Cohesion Index (SCI). Source: European Social Survey

Certainly, using such demanding criteria leads to the dropping of many relevant items which could be useful in the construction of an SCI. However, not doing so would make it impossible to construct an integrated measure that can capture SC at different social levels as proposed. Contemporary public opinion surveys such as the ESS make it possible to observe these items not only as a country aggregate, but also across individuals and different social categorisations within societies, allowing us thereby to operationalise SC at different social levels. The survey is conducted in 38 European societies on a bi-annual basis, starting in 2002 and going up to 2018, covering more than 400,000 individuals (Table 3 in "Appendix"). After screening all questionnaires of the survey (face validity) in search of interchangeable items which may indicate the three dimensions more concretely, three items for social trust (V1–V3), five items for institutional trust (V4–V8), and three items for openness (V9-V11) are selected. The chosen items (Fig. 2) are all variables measured on a continuous scale (Table 4 in "Appendix").

To understand how each of the 11 observed variables relates to the three latent components, we first need to turn to a *reflective* measurement approach. In a reflective measurement model a latent construct such as ‘social trust’ is said to be reflected in the individually observed attitudes of trustworthiness, helpfulness and fairness. The observed indicators should ideally form a “unidimensional reflection of the latent indicators” (Bollen & Lennox, 1991: 308). To validate all three separate measurement models of the dimensions, the built-in Structural Equation Modelling (SEM) tool in Stata 16 is used. Additionally, a covariation term between each of the three latent dimensions is added and is represented by the double-headed arrows. The factor loadings are standardised to enable an easier interpretation. This method is also used in order to scale the latent variables when running a confirmatory factor analysis which allows us to view the relative factor loading of each variable in comparison to the others. The individual factor loadings can be thought of as standardised regression coefficients. The confirmatory factor analysis is carried out at the individual-level, with a total of 374,378 individual observations (Table 7 in "Appendix"). To handle the missing data, I run a maximum likelihood (ML) estimation accounting for missing values. ML estimates are both more consistent and more efficient than pair-wise or list-wise estimation, yielding more unbiased results (Byrne, 2010: 356). A factor loading of 0.20 and above is considered as allowing a sufficient measurement of a latent variable (Peterson, 2000: 272).

**Fig. 3:**
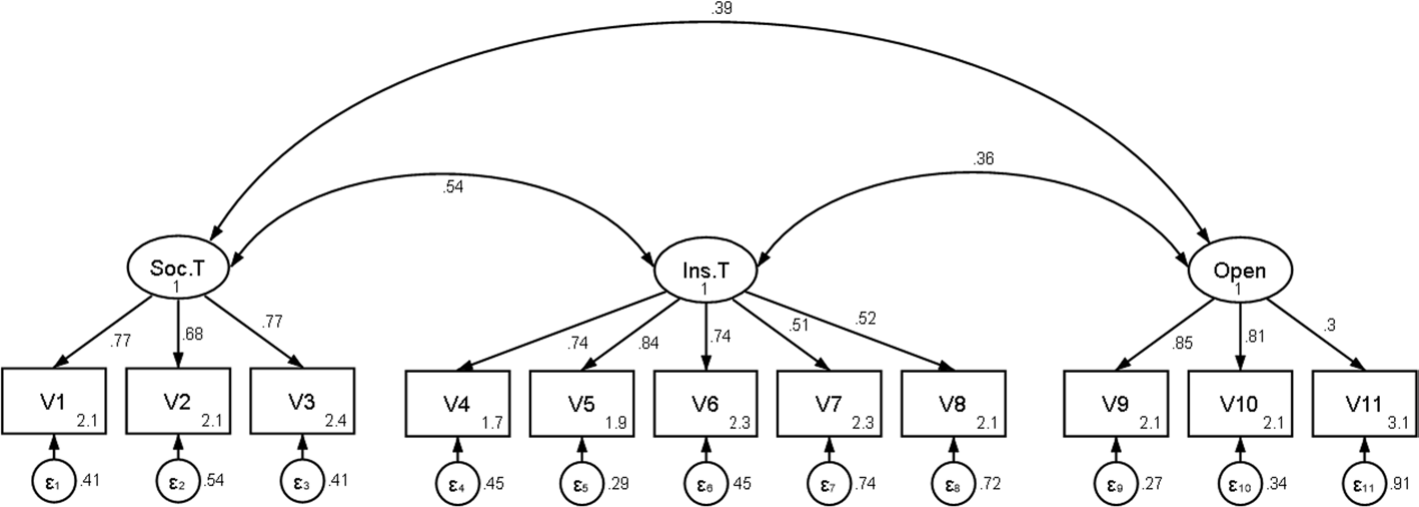
Confirmatory factor analysis for social trust, institutional trust and openness. *Note*: Soc.T = Social Trust; Ins.T = Institutional Trust; Open = Openness

The models show that the factor loadings are all highly significant (Fig. 3). The factor loading of V11 is lower at 0.3. And it will be weighted as such when extracting the final *factor score* of the openness dimension. The covariation terms reveal a strong correlation at the individual-level between social trust and institutional trust at 0.54; between social trust and openness at 0.39; and between institutional trust and openness at 0.36. Thus, it can be validated that the dimensions are overall strongly related to each other, even at the level of individual orientations in all societies, taken together with a very large sample size. The three dimensions will later be added together in a formative/composite construct. While Langer et al., (2017: 326) use also a formative construct for their index, they propose that a criteria for a good formative measure is that the domains of SC must be independent from each other (Langer et al., 2017: 326). According to them, the dimensions must exhibit a low correlation to each other. However, contrary to reflective models, in a formative measurement model, we do not have anything to say about the covariances of items, which could be zero, positive or negative (Hardin, 2017: 5). Since we are dealing with a theoretically constructed measure, “items can have any pattern of intercorrelation” (Bollen & Lennox, 1991: 307). Although the components do not reflect a single underlying property but rather different qualities, they can nonetheless be correlated without undermining the validity of the new combinatory construct. Only because the individual dimensions are correlated to each other, it does not mean that they are also interchangeable qualities.

Many current studies on SC, do not provide adequate and updated ‘goodness-of-fit’ tests of their proposed measurement models (see Dragolov et al., 2016: 28; Langer et al., 2017: 333). To validate such a measurement theory, a couple of such robustness checks are needed (Fig. 7 in "Appendix"). Goodness-of-fit models using SEM methods for assessing reliability of items are considerably superior to some older methods. The primary task in this model-testing procedure is to determine the goodness-of-fit between the hypothesised model and the sample data. The chi-square value indicates a good fit of the model. However, this is a rather outdated measure of goodness-of-fit and is known to be affected by sample size. The root mean squared error of approximation (RMSEA), which is a much better indicator of goodness-of-fit, is below 0.08, indicating a good fit of the model to the data (Kelley & Lai, 2011: 2). The “pclose” value indicates that the model does not deviate significantly from a close fit. The comparative fit index (CFI), and the Tucker–Lewis index (TLI) are both above 0.9 indicating a very close fit of the model (Xia & Yang, 2019: 409). The coefficient of determination (CD) of 0.99 indicates an exceptionally good fit. Altogether, the measurement model can be considered statistically validated and the measurement hypothesis can thus be confirmed.

The last step of the procedure is the creation of the *formative* (i.e., combinatory) social cohesion index (SCI). The confirmatory factor analysis produces factor scores for all three dimensions with a mean of zero but with different standard deviations. To be able to combine the three components into one overall index, the three dimensions need to be normalised to be on the same scale of measurement (i.e., var–min value/max value–min value). After this step, the following aggregation method for the final SCI is used: the three resulting proportions are given equal weights in aggregating them into the index. Thus, each constitutive component is ‘weighted’ as one third (1/3) of the overall SCI. The SCI is thus also a normalised measure ranging from zero to one, with zero corresponding to a theoretically non-cohesive value and one corresponding to a highly cohesive value. However, one and the same SCI score of two individuals can mean that these individuals score quite differently on the three constative dimensions. This combinatory logic prescribes “compositional substitutability” among partial responses (Goertz, 2006: 11). According to Welzel and Inglehart (2016: 1075) when using such a composite indicator “variability in the composition of partial responses does not affect how an overall response relates to its expected antecedents and consequences.” Since the SCI can be composed and decomposed, it allows us to do both: identify individuals or societies with different variability in terms of cohesion profiles (i.e., ‘cohesion regimes’), that is the “internal convergence” of items (Welzel & Inglehart, 2016: 1075), as well as map the combinatory indicator independently to its supposed antecedents and consequences, that is the “external validity” of the combinatory construct. Thus, at the individual level the new measure allows one to identify individuals with different SC profiles and at the societal level it allows one to identify societies with different social cohesion "regimes", while at the same time allowing us to validate the external linkage of the combinatory SCI when analyzing it in relation to its antecedents and consequences. This point will be illustrated again in the results section below.

Consequently, we obtain a final SCI value for a total of 374,378 individuals in the survey. We can additionally obtain the same separate scores for the three individual dimensions which can also be observed in isolation. The SCI and its components can be observed directly at the individual-level. They can further be aggregated across defined social categorisations as well as across entire societies. As such, the index allows us to observe different patterns and profiles at the individual-, group- and country-level, as well as make inferences about these levels’ interdependence. It allows us to use individual-level orientations of SC as direct outcomes of other relevant antecedents. At the so-called ‘meso-level’ it allows us to identify large intergroup differences in orientations of SC within societies indicating latent conflict. Finally, we can aggregate these orientations at the level of societies, making it possible to compare societies to each other and classify them in different clusters. Additionally, as an aggregate, the SCI can be used as an antecedent in its own right, to see if the overall degree of SC of a society has a substantial effect on other desirable outcomes, over and above other relevant factors such as affluence or inequalities. The strength of this theoretically-derived measure will be illustrated below by touching upon a highly relevant debate, namely the link between inequalities, SC and subjective well-being.

## Inequalities, Cohesion and Well-Being: The Path of Action

In an extensive effort to define SC and set it apart from its antecedents and consequences, Schiefer and Noll come to the conclusion that “equality, cohesion, and quality of life can be put in a causal chain.” Hence, “a cleavage between the poor and the rich might weaken cohesion due to perceived deprivation and inequality”, and in turn “stronger societal cohesion might contribute to the well-being of the society’s members.” This chimes with multiple earlier studies that find that social cohesion is generally weaker and develops more slowly in highly-stratified societies (Pickett & Wilkinson, 2010: 107; Rothstein & Uslaner, 2005: 41). Vergolini (2011: 207) finds that while stratification factors have a “stronger impact on network density, the economic inequality more deeply affects the dimension of civic integration.” On the other hand, the beneficial effects of societal cohesion on subjective well-being have also been addressed before (Delhey & Dragolov, 2016: 163; Klein, 2013: 891). Yet, this “causal chain” is never fully empirically disentangled and analysed in a path of action. The very assessment by Schiefer and Noll, that inequalities affect members ‘perceived’ situations, while ‘societal’ cohesion contributes to the well-being of individual members, posits a crucial mechanism, which they do not account further for: as an outcome of inequalities, SC should be measured at the level of individual orientations, while as a predictor of well-being, SC should be measured as an aggregate. This analysis seeks to empirically validate this chain of action.

Methodologically, this initially involves using multi-level regression models with the scores of the SCI, and separately for each of its components, as dependent variables at the individual-level. The predictors of these orientations can be both contextual macro-scores as well as individual characteristics. In a three-level model, individual orientations of SC are ‘nested’ in a given country-wave, with the country-wave observations then nested in the individual countries (Appendix A.7). The individual-level predictors of age, gender, education, income, employment status and self-rated discrimination all derive from the ESS survey (Table 1). The post-communist society variable is coded as a binary variable for each society. The Gini coefficient for disposable income derives from the Standardized World Income Inequality Database (SWIID), which seeks to maximise the comparability of income inequality estimates for the broadest possible coverage of countries and years (Solt, 2020: 1183). The Gender Inequality Index (GII) reflects gender-based disadvantage and the loss in potential human development due to inequalities in female and male resources (UNDP, 2016). The welfare expenditure variable captures expenditure on health and education, and is a proxy obtained from the World Bank’s “World Development Indicators” (World Bank, 2016). GDP per capita estimates, known to strongly affect other developmental outcomes, are also retrieved from the same dataset. Since we are dealing with unstandardised regressions, a descriptive account of these variables and their scaling can give more insight into the substantive impact of each independent variable (Table 8 in "Appendix").

**Table 1:** Multi-level regression of inequalities on individual orientations of social cohesion

	(1) SCI		(2) SOC.T		(3) INS.T		(4) OPEN	
	Coef	SE	Coef	SE	Coef	SE	Coef	SE
*Level: Individual*								
Age (years)	− 0.0002***	(0.0001)	0.0002	(0.0001)	− 0.0001*	(0.0001)	− 0.0007***	(0.0001)
Gender (women)	0.0049**	(0.0023)	0.0081***	(0.0023)	− 0.0035	(0.0022)	0.0098**	(0.0039)
Education (years)	0.0057***	(0.0005)	0.0046***	(0.0004)	0.0031***	(0.0006)	0.0102***	(0.0007)
Income (difficulty)	− 0.0304***	(0.0016)	− 0.0323***	(0.0015)	− 0.0333***	(0.0018)	− 0.0256***	(0.0019)
Unemployed	− 0.0058	(0.0044)	− 0.0088*	(0.0046)	− 0.0100*	(0.0060)	0.0055	(0.0048)
Discriminated	− 0.0355***	(0.0040)	− 0.0497***	(0.0035)	− 0.0613***	(0.0051)	0.0060	(0.0066)
*Level: Country-wave*								
Income inequality (Gini)	− 0.0027***	(0.0006)	− 0.0022***	(0.0004)	− 0.0036***	(0.0014)	− 0.0022**	(0.0011)
Gender inequality (GII)	− 0.0006	(0.0006)	0.0001	(0.0007)	− 0.0008	(0.0008)	− 0.0009	(0.0006)
Welfare expenditure	0.0000**	(0.0000)	0.0000	(0.0000)	0.0000	(0.0000)	0.0000	(0.0000)
GDP p.c	0.0012***	(0.0004)	0.0012**	(0.0005)	0.0019*	(0.0011)	0.0002	(0.0008)
Post-communist	− 0.0307	(0.0227)	− 0.0463	(0.0291)	− 0.0424	(0.0302)	− 0.0144	(0.0261)
Constant	0.5744***	(0.0207)	0.5469***	(0.0256)	0.6187***	(0.0559)	0.5170***	(0.0364)
Variance (countries: cons.)	0.0019***	(0.0005)	0.0032***	(0.0008)	0.0032***	(0.0009)	0.0032***	(0.0008)
Variance (country-waves)	0.0003***	(0.0001)	0.0002***	(0.0000)	0.0009***	(0.0002)	0.0007***	(0.0001)
Variance (residuals)	0.0167***	(0.0005)	0.0238***	(0.0009)	0.0259***	(0.0010)	0.0329***	(0.0012)
N (countries)	36		36		36		36	
N (country-waves)	219		219		219		219	
N (individuals)	346,590		346,590		346,590		346,590	
Log Likelihood	177,149.7		125,983.3		115,659.5		93,026.36	
AIC	− 354,271.4		− 251,938.6		− 231,291.1		− 186,024.7	
BIC	− 354,123.9		− 251,791.1		− 231,143.6		− 185,877.3	

The table shows unstandardized regression coefficients with standard errors in parenthesis. The models incorporate available sample weights

Significance: *p < .10, **p < .05, ***p < .01

The models show that age and gender have a significant, albeit substantively very small effect on SC orientations. The degree of education (years of schooling) has a significantly positive effect particularly on orientations of openness, confirming that education allows for “better cross-cultural understanding and more effective civic participation” (Green et al., 2003: 460). Self-reported unemployment and discrimination have a particularly strong negative impact on orientations of social and institutional trust. Surprisingly, neither unemployment nor being a member of a discriminated-against group has an effect on orientations related to openness. Unemployed or discriminated-against individuals are therefore not necessarily less open to outgroups than other individuals, who are not in such disadvantaged positions. Having difficulty in making ends meet is a highly significant negative predictor on the SCI, and all its components. Clearly, individual resources strongly affect cohesion orientations. The contextual control of income per capita has a positive impact on the SCI, and on all but the openness dimension. Thus, country affluence alone may not be sufficient to achieve more openness in a society. Being a post-communist society has no significant effect once the other factors are accounted for. Neither does the gender inequality index or the index on welfare expenditure, once income inequality as measured by the Gini coefficient are taken into account, which has a strong negative impact on orientations of SC. Income inequality has a strongly negative effect on institutional trust in particular, as well as on social trust and openness. The significant association with openness can be considered evidence that inequalities also harm cross-group relations in society. The method provides us with real hints about the possible mechanisms at play, through which SC orientations are affected more specifically. Both individual and context factors related to inequalities can be said to affect the individuals’ situation, which in turn affects how they form attitudes with regard to SC.

**Fig. 4:**
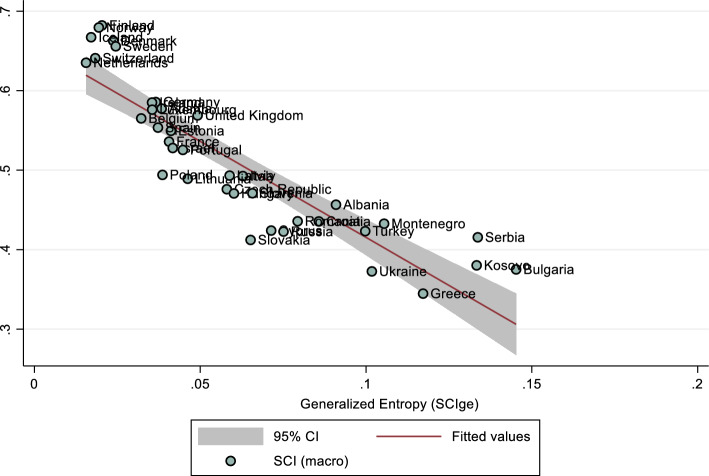
Entropy of SCI orientations as compared to country means

Welzel and Inglehard have described a “coherence inducing force” when observing how a country’s cognitive mobilisation leads to greater coherence of emancipatory values within a society (Welzel & Inglehart, 2016: 1080). In the interaction between inequalities and orientations of social cohesion, exactly the inverse relationship could be claimed: as material and perceived inequalities increase, so does the dissimilarity (i.e., incoherence) of SC orientations between members within a society. To test this, let us plot the ‘generalised index of entropy’ (Jenkins, 2021) which considers the mean log deviation of individual orientations of SC within a society, and which we may denote as ${SCI}_{ge}$. Similar indices include the Atkinson Inequality Measure (Atkinson, 2005: 245), and Sen's (1976: 20) welfare measure. Basically, the higher the generalised index of entropy, the more unequal the individual orientations of SC within a society will be. We can extract and illustrate the general entropy of individual SCI orientations for each society and compare this entropy to the aggregate country mean index for the last observable wave in which a society is present (Fig. 4). From these statistics, we can already gain some very substantial insights. For one, we observe that the most cohesive societies in the sample exhibit the lowest entropy of cohesion orientations. The finding is consistent and the overall correlation between entropy in cohesion orientations and country-mean societal cohesion for all waves is highly negative (r = −0.95, N = 231). This systemic and almost linear association between dissimilarities in individual orientations and country means of cohesion can hardly be classified as erratic.

**Fig. 5:**
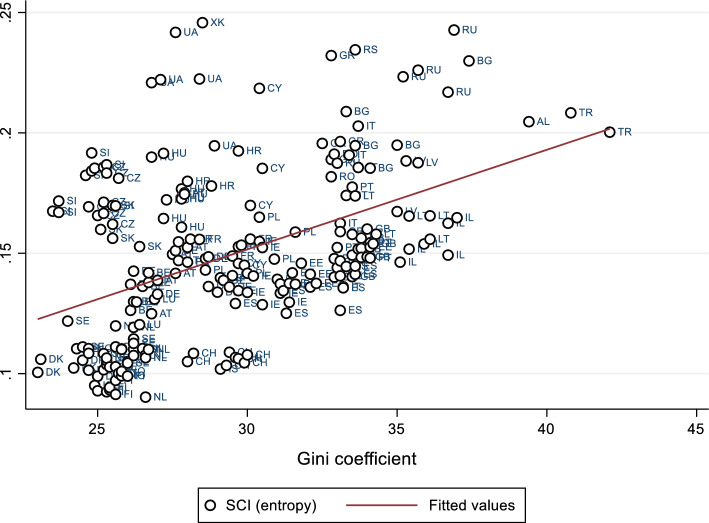
Linear regression scatterplot of Gini coefficient of income inequalities and entropy of cohesion orientations

Additionally, in a pair-wise correlation, we see that macro-level Gini inequality scores and the overall entropy of cohesion are strongly associated (r = 0.55, *p* < 0.05. pairs: 203). In a lagged multiple regression model, income inequalities in earlier waves still significantly affect later waves of SCI entropy, even when controlling for earlier waves of income and education (Table 9 in "Appendix"). Thus, the more income inequities in a society in earlier waves, the more dissimilar we can expect individual orientations of SC to be in later waves. In more unequal societies, generally greater differences in individual orientations of SC will develop. We can visualise this macro-relationship with a scatterplot depicting the linear regression between differences in income inequality (x) and the general entropy in cohesion orientations in the individual countries. Considering all country-wave observations in the sample we notice that although there are outliers, generally the higher the levels of income inequality, the more dissimilar orientations of SC will become (Fig. 5). The finding is able to show that the measure created using this conception of SC performs very well in terms of its external validity (Welzel & Inglehart, 2016: 1075).

The degree to which SC orientations are shared, provides essential insight into the overall performance of the SCI. Using this index, this sort of dissimilarity can also be computed for select social categorisations (e.g., ethnic & religious communities, regions, social classes, gender, migration background etc.) within societies. It is here that observing the ‘meso-level’ becomes very insightful. Across these categories within societies, major differences in the orientation of cohesion may arise because of ‘horizontal’ (Stewart, 2007) or ‘categorical’ inequalities, where differences in access to resources and opportunities may vary “systematically with membership in social categories” (Massey, 2007: 5). Furthermore, social-psychological research concerned with “frames of reference”, “reference cognition”, “relative deprivation”, and the “emotional resentment” of individuals and groups (Cropanzano & Folger, 1989: 293; Folger, 1987: 183; Rose, 2006: 1; Salmela & von Scheve, 2017: 567), can provide additional insight into how groups’ differences in orientations emerge and what their impact on cohesion might be. For instance, regions within societies as a social categorisation with their local governments are tremendously important geopolitical entities, especially in the European context. Importantly, not many studies look at the interdependence of cross-regional inequalities and societal—that is ‘macro’—cohesion. The ESS includes a quite substantial amount of information on regional units. Thus, the sample size encompasses a total of 348 regional unit within 29 European societies (for the last observable country-wave), for which sufficient information on the regional-level can be gathered.

**Fig. 6:**
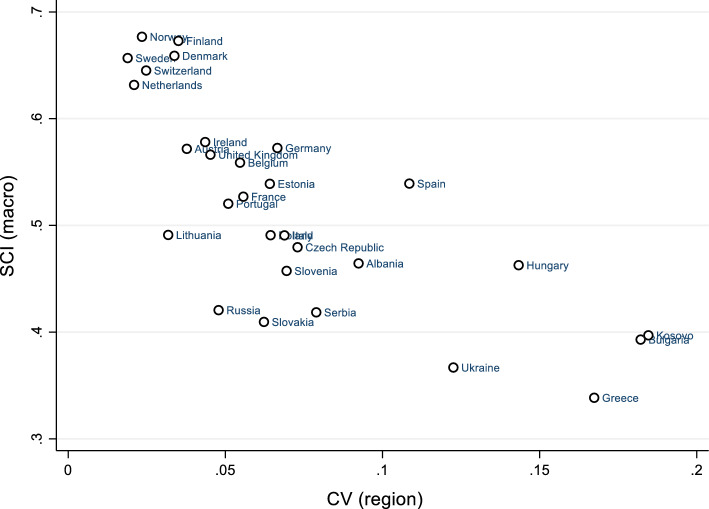
Aggregate SCI as compared to the regional dissimilarity of orientations of cohesion (CV)

**Fig. 7:**
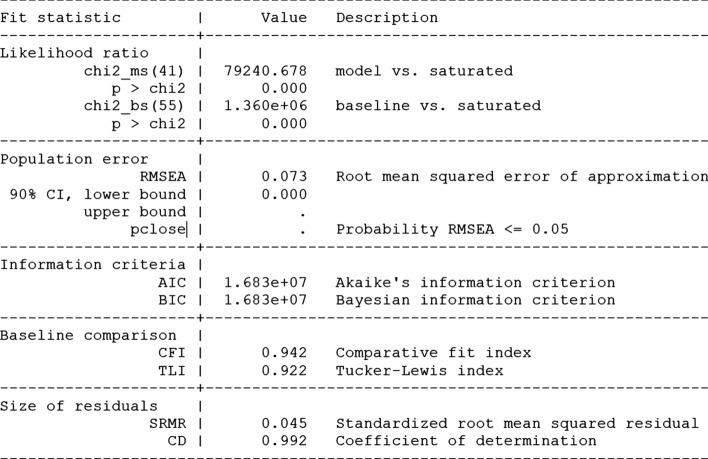
Goodness-of-fit statistics of confirmatory factor analysis

While looking at each individual regional unit of each society is beyond the scope of this study, we can instead look at the coefficient of variation (CV) in cross-regional orientations of cohesion by using this data and thereby assess the regional dissimilarity of SC for each society (Fig. 6). The coefficient of variation (CV) is also called a relative standard deviation (Appendix 10), and is a standardised measure of dispersion, expressed as the ratio of the standard deviation to the mean (Everitt & Skrondal, 2010: 89). Langer et al., (2017: 328) have employed a similar measure to investigate differences across ethnic groups in the context of 17 African societies. In the example used here, the correlation between the regional CV and aggregate SCI is highly negative and significant, even when considering all country-wave observation for which the regional CV can effectively be obtained (r = −0.75; *p* < 0.01; pairs: 115). Generally, as the regional CV increases, the overall degree of SC in society decreases. Overall, the CV values largely affirm known regional divisions and hence latent conflict in these societies, particularly in the case of Kosovo (Yannis, 2001: 41), Greece (Coccossis & Psycharis, 2008: 135), Spain (Bieri, 2014: 1), Ukraine, Bulgaria and Hungary. According to some European scholars, large regional disparities have led to “the emergence of a highly educated and internationally successful professionals and entrepreneurs located mainly in metropolitan areas on the one hand, and structural unemployment, persistent poverty and social exclusion in peripheral regions on the other” (Smętkowski, 2013: 1529). This seems to suggest that regional dissimilarity in SC orientations arises as a consequence of material inequalities faced by members of these regions. Income inequalities alone may not explain entirely this dissimilarity, as regional differences overlap in practice with other aspects of differentiation and latent conflict such as based on ethnicity, religion etc. Another insightful finding is that regions remain mostly dissimilar in their orientations of institutional trust (Table 10 in "Appendix"). This is particularly the case in Bulgaria, Albania, Kosovo, Ukraine, Greece, Hungary and Spain where the coefficient of variation for institutional trust is considerably higher than 0.15. Political divisions between regions and conflicts over political power can be said to significantly hamper societal cohesion there. These finding are more insightful when we consider that at the individual-level it was orientations of institutional trust in particular that were negatively affected by income inequalities.

**Fig. 8:**
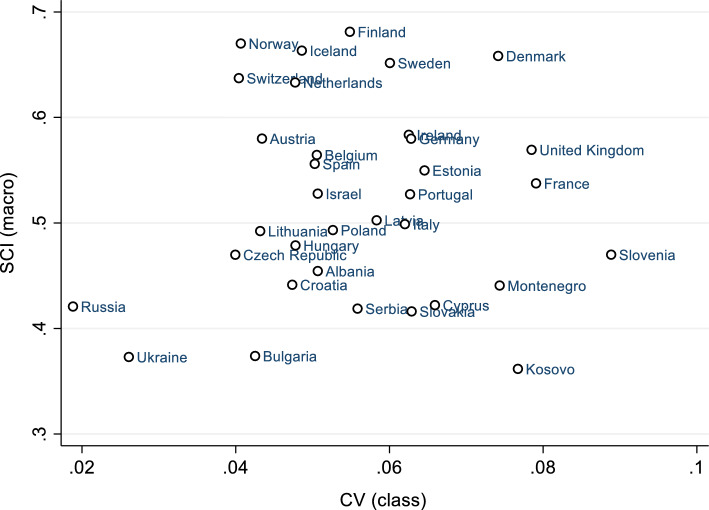
Coefficient of Variation for the dispersion of SCI across SES as compared to the overall degree of societal cohesion

Importantly, the SCI allows us to investigate which societies exhibit higher dissimilarities, and on which categorisations these dissimilarities are based on. The prominent Bertelsmann Cohesion Radar (Dragolov et al., 2016) cannot account for such dissimilarities. For instance, using the five-group socio-economic status (SES) schema proposed by Oesch (2006: 263), it is possible to reproduce the very same plot comparing the dissimilarity of orientations across SES groups within a society to its overall country mean (Fig. 8 in "Appendix"). However, in this case, we see that the within-country dissimilarities based on SES categories are not negatively related to overall societal SC. The correlation between the CV for SES groups and aggregate SCI when considering all country-waves is neglectable and statistically insignificant (r = −0.01; *p* = 0.87; pairs = 100). Thus, the findings seem to suggest that certain cleavages have stronger potential to produce latent conflict and impair societal cohesion than others. At least in the European context, it can be said that regional cleavages are more salient than those based on socio-economic status.

**Fig. 9:**
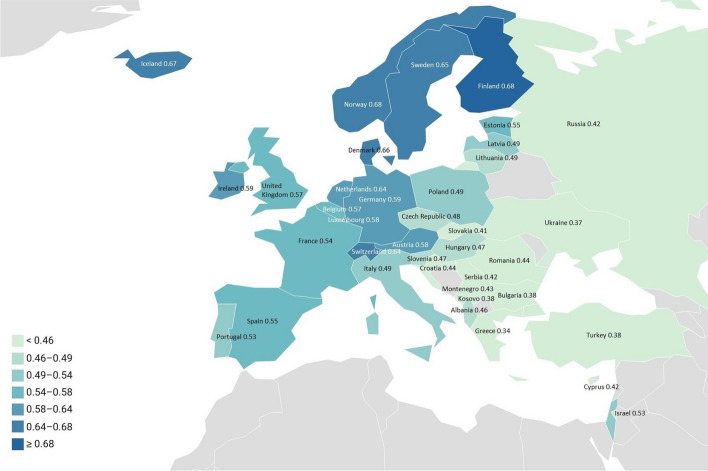
Aggregate social cohesion in European societies

**Fig. 10:**
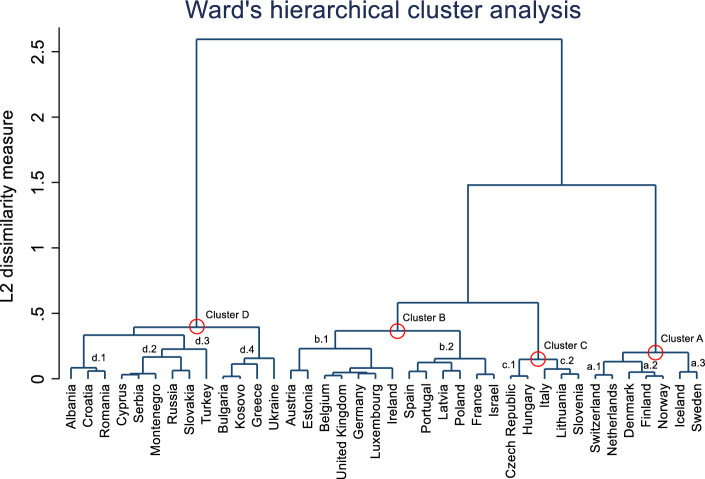
Dendrogram of Ward’s Linkage hierarchical cluster analysis for social trust, institutional trust and openness

**Fig. 11:**
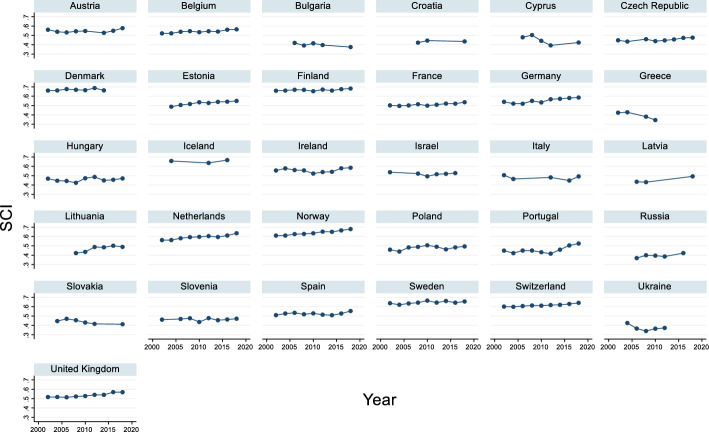
Change of societal cohesion in time

Finally, we can observe the societal aggregate levels of SC. First, we can generate a choropleth map of the aggregate SCI score for the last observable wave, comparatively for each society (Fig. 9 in "Appendix"). An intuitive picture emerges with the Scandinavian and smaller European societies exhibiting the highest levels of SC and the south-eastern European societies scoring the lowest. To observe also the “internal convergence” (Welzel & Inglehart, 2016: 1075) of items and whether the individual three components point to meaningful patterns and clusters across cases, which would make it plausible to group them into certain categories or “cohesion regimes” (Dickes & Valentova, 2013: 842; Dragolov et al., 2016: 51; Green & Janmaat, 2011: 118), we can perform a hierarchical cluster analysis (Ward, 1963: 237), showing the macro-similarities of societies in the three respective components of social trust, institutional trust and openness (Fig. 10 in "Appendix"). Four such meaningful patterns emerge. Additionally, we can plot the SCI and its components over time to observe any temporal changes (Fig. 11 in "Appendix"). We can make inference about what might have driven this change. As it is beyond the scope of this paper to analyse all these descriptive statistics in detail, they remain to be addressed in a follow-up study.

At the end of this analysis, I shall focus only on the additional question posed initially, namely—does the aggregate degree of SC in the sense of a societal antecedent have an impact on individual subjective well-being (SWB)? If the macro-SCI has a significant effect on self-reported SWB over and above individual traits as well as other important societal factors such as wealth and inequalities, then such a link can indeed be also empirically established. The evidence from the multi-level regression models suggests that aggregate SC as a societal condition has without a doubt a very strong and significant effect on SWB as an individual-level outcome (Table 2).

**Table 2:** Multi-level regression of societal cohesion on individual subjective well-being

	(1) SWB		(2) SWB		(3) SWB		(4) SWB	
	Coef	SE	Coef	SE	Coef	SE	Coef	SE
*Level: Individual*								
Age (years)	0.0002**	(0.0001)	0.0002**	(0.0001)	0.0002**	(0.0001)	0.0002**	(0.0001)
Gender (women)	0.0136***	(0.0015)	0.0136***	(0.0015)	0.0136***	(0.0015)	0.0136***	(0.0015)
Education (years)	− 0.0005**	(0.0003)	− 0.0005**	(0.0003)	− 0.0005**	(0.0003)	− 0.0005**	(0.0003)
Income (difficulty)	− 0.0636***	(0.0029)	− 0.0636***	(0.0029)	− 0.0636***	(0.0029)	− 0.0637***	(0.0029)
Unemployed	− 0.0361***	(0.0040)	− 0.0361***	(0.0040)	− 0.0361***	(0.0040)	− 0.0361***	(0.0040)
Health (self-rated)	0.0576***	(0.0017)	0.0576***	(0.0017)	0.0576***	(0.0017)	0.0576***	(0.0017)
Discriminated	− 0.0495***	(0.0034)	− 0.0495***	(0.0034)	− 0.0495***	(0.0034)	− 0.0495***	(0.0034)
*Level: Country-wave*								
SCI (macro)	0.4005***	(0.0744)						
Income inequality (Gini)	0.0002	(0.0008)	0.0010	(0.0009)	− 0.0008	(0.0011)	− 0.0005	(0.0009)
GDP p.c	0.0005	(0.0004)	0.0004	(0.0003)	0.0008*	(0.0004)	0.0012***	(0.0005)
Post-communist	− 0.0066	(0.0104)	− 0.0113	(0.0098)	− 0.0094	(0.0118)	− 0.0210	(0.0132)
SOC.T (macro)			0.4354***	(0.0792)				
INS.T (macro)					0.2168***	(0.0548)		
OPEN (macro)							0.1717***	(0.0592)
Constant	0.3857***	(0.0469)	0.3512***	(0.0540)	0.5019***	(0.0367)	0.5053***	(0.0419)
Variance (countries: cons.)	0.0005***	(0.0001)	0.0006***	(0.0001)	0.0006***	(0.0002)	0.0007***	(0.0002)
Variance (country-waves)	0.0002***	(0.0000)	0.0002***	(0.0000)	0.0003***	(0.0000)	0.0003***	(0.0000)
Variance (residuals)	0.0260***	(0.0012)	0.0260***	(0.0012)	0.0260***	(0.0012)	0.0260***	(0.0012)
N (countries)	36		36		36		36	
N (country-waves)	229		229		229		229	
N (individuals)	398,878		398,878		398,878		398,878	
Log Likelihood	161,160.4		161,162.9		161,148		161,140.3	
AIC	− 322,290.8		− 322,295.9		− 322,266		− 322,250.6	
BIC	− 322,127.3		− 322,132.4		− 322,102.6		− 322,087.1	

The table shows unstandardized regression coefficients with standard errors in parenthesis. The models incorporate available sample weights

Significance: *p < .10, **p < .05, ***p < .01

Although all components of SC have a significant effect on SWB, it is evident that social trust has the strongest substantive effect, confirming previous studies that have established this link (Bjørnskov, 2008: 54; Helliwell & Wang, 2006: 1). In fact, once we include SC and its components in the models, the effect of income inequalities on SWB diminishes completely and the relationship between the Gini coefficient and SWB becomes insignificant. Likewise, the positive effect of national affluence on SWB as measured by GDP p.c. weakens substantially. Thus, scores on the SCI can be confidently said to be the most significant macro-level predictor of individual SWB over and above the effects of inequalities and income. However, difficulty in making ends meet has a significant negative effect on the SWB of individuals. The findings here suggest that, in fact, it is relative income and not absolute income that matters most to SWB. In the Bertelsmann Cohesion Radar study, the authors establish a strong link between macro-level cohesion and aggregate SWB across countries (Dragolov et al., 2016: 65). However, SWB can, by definition, hardly be considered a societal-level variable. Aggregating SWB to the level of societies misses the importance that micro-level predictors such as health, education and income can have on an individual’s self-rated level of SWB. These individual factors are all found to have a strong effect on SWB respectively. One surprising finding that is worth mentioning here is that degree of education seems to have a negative effect on SWB, which may be considered a counterintuitive finding. However, reports of this negative association are not rare, but are rarely clarified. Kristoffersen (2018: 64) points out that this relationship is in fact “consistent with the idea that education is associated with higher expectations with respect to life circumstances. Consequently, education may be associated with greater subjective well-being only insofar as the ability to meet (or exceed) expectations is improved.” We could use the aggregate SCI in a similar fashion to see if it effect on other outcomes such as for instance the social mobility of members, their human capabilities (Lanzi, 2011: 1089), and sense of safety (Hirschfield & Bowers, 1997: 1275).

## Conclusion

The study has tackled a highly important concept and has sought to provide a clear and novel approach to the conceptualisation and measurement of SC. An additional framework was necessary because current analytical approaches used today are insufficient. The contribution and research potential lays primarily in the conceptual reformulation and methodological advances made here. First, a plausible way to reconcile individualistic and holistic explanations of SC, which do not need to be antagonistic, was presented. The approach ascribes a logical directionality to SC. In this view, explanations of a collective phenomenon and a social fact, such as SC must be supplied with more ‘micro’ foundations that specify an action-theoretic mechanism. The conception provides that SC is manifested in individual attitudes, without excluding that when shared by collectives, the association of these attitudes constitutes a social force in its own right. Importantly, an effective method of measuring SC at different levels as both consequence and antecedent of other social phenomena, albeit as an integrated whole was presented. The proposed method was able to show the interdependence of these levels (e.g., regional to societal) in the case of SC. Adding to the debate of “misconceptions of measurement equivalence” (Welzel & Inglehart, 2016: 1068), the SCI is able to provide without a compromise the necessary information to observe distinctly the internal convergence as well as the external validity of measurement items.

There is a limit to the potential empirical realisation of the data in this particular study. But the constructed SCI provides a host of other possibilities, such as the application of interaction effects at the individual-levels; inference on various other social categorisation (regions, classes, languages, etc.) at the meso-level; and a variety of statistics at the macro-level which convey a more up-to-date assessment of SC in a larger and more diverse sample of contemporary European societies. A more nuanced profile of SC in these societies than currently found is certainly possible. With regard to the link between inequalities, SC and SWB, the study is able to confirm that individual orientations of SC (and their dissimilarity) are significantly affected by inequalities, while SWB is significantly affected by the degree of societal cohesion over and above inequalities and other relevant factors. This highly-cited relationship is distinctly analysed here in a path of action at three different levels. The findings provide more insight as to how cohesion is affected at the individual-level, and how in turn it has a predicative power in its own right when aggregated to the level of collectives. The analysis at the ‘meso-level’ provides additional insights into the internal within-country social dynamics and cleavages—in this case regional—that can influence societal level cohesion.

## Appendix

See Tables 3, 4, 5, 6, 7 and Fig. 7.

**Table 3:** Tabulation of individual observations by country and survey rounds

Country	ESS round									
	1(2002)	2(2004)	3(2006)	4(2008)	5(2010)	6(2012)	7(2014)	8(2016)	9(2018)	Total
Albania	0	0	0	0	0	1201	0	0	0	1201
Austria	2257	2256	2405	2255	2259	0	1795	2010	2499	17,736
Belgium	1899	1778	1798	1760	1704	1869	1769	1766	1767	16,110
Bulgaria	0	0	1400	2230	2434	2260	0	0	2198	10,522
Croatia	0	0	0	1484	1649	0	0	0	1810	4943
Cyprus	0	0	995	1215	1083	1116	0	0	781	5190
Czech Republic	1360	3026	0	2018	2386	2009	2148	2269	2398	17,614
Denmark	1506	1487	1505	1610	1576	1650	1502	0	0	10,836
Estonia	0	1989	1517	1661	1793	2380	2051	2019	1904	15,314
Finland	2000	2022	1896	2195	1878	2197	2087	1925	1755	17,955
France	1503	1806	1986	2073	1728	1968	1917	2070	2010	17,061
Germany	2919	2870	2916	2751	3031	2958	3045	2852	2358	25,700
Greece	2566	2406	0	2072	2715	0	0	0	0	9759
Hungary	1685	1498	1518	1544	1561	2014	1698	1614	1661	14,793
Iceland	0	579	0	0	0	752	0	880	0	2211
Ireland	2046	2286	1800	1764	2576	2628	2390	2757	2216	20,463
Israel	2499	0	0	2490	2294	2508	2562	2557	0	14,910
Italy	1207	1529	0	0	0	960	0	2626	2745	9067
Kosovo	0	0	0	0	0	1295	0	0	0	1295
Latvia	0	0	1960	1980	0	0	0	0	918	4858
Lithuania	0	0	0	2002	1677	2109	2250	2122	1835	11,995
Luxembourg	1552	1635	0	0	0	0	0	0	0	3187
Montenegro	0	0	0	0	0	0	0	0	1200	1200
Netherlands	2364	1881	1889	1778	1829	1845	1919	1681	1673	16,859
Norway	2036	1760	1750	1549	1548	1624	1436	1545	1406	14,654
Poland	2110	1716	1721	1619	1751	1898	1615	1694	1500	15,624
Portugal	1511	2052	2222	2367	2150	2151	1265	1270	1055	16,043
Romania	0	0	2139	2146	0	0	0	0	0	4285
Russia	0	0	2437	2512	2595	2484	0	2430	0	12,458
Serbia	0	0	0	0	0	0	0	0	2043	2043
Slovakia	0	1512	1766	1810	1856	1847	0	0	1083	9874
Slovenia	1519	1442	1476	1286	1403	1257	1224	1307	1318	12,232
Spain	1729	1663	1876	2576	1885	1889	1925	1958	1668	17,169
Sweden	1999	1948	1927	1830	1497	1847	1791	1551	1539	15,929
Switzerland	2040	2141	1804	1819	1506	1493	1532	1525	1542	15,402
Turkey	0	1856	0	2416	0	0	0	0	0	4272
Ukraine	0	2031	2002	1845	1931	2178	0	0	0	9987
United Kingdom	2052	1897	2394	2352	2422	2286	2264	1959	2204	19,830
Total	42,359	49,066	47,099	61,009	54,717	54,673	40,185	44,387	47,086	440,581

*Source*: Europe social survey (2002–2018), own calculation

**Table 4:** Coding and observations for individual variables

Variable	Obs	Mean	Std. Dev	Min	Max
(V1)	437,427	4.964	2.482	0	10
(V2)	436,620	4.838	2.377	0	10
(V3)	434,732	5.534	2.347	0	10
(V4)	426,741	4.341	2.621	0	10
(V5)	427,074	5.063	2.717	0	10
(V6)	433,386	5.907	2.643	0	10
(V7)	416,781	5.525	2.387	0	10
(V8)	433,398	5.258	2.597	0	10
(V9)	417,640	5.438	2.588	0	10
(V10)	415,870	4.836	2.354	0	10
(V11)	418,832	3.715	1.231	1	5

V11 is coded inversely and was reversed

**Table 5:** Individual level pairwise correlations of all variables

Variables	(V1)	(V2)	(V3)	(V4)	(V5)	(V6)	(V7)	(V8)	(V9)	(V10)	(V11)
(V1)	1.000										
(V2)	0.515***	1.000									
	(0.000)										
(V3)	0.590*****	0.534*****	1.000								
	(0.000)	(0.000)									
(V4)	0.334*****	0.288*****	0.303*****	1.000							
	(0.000)	(0.000)	(0.000)								
(V5)	0.334*****	0.298*****	0.309*****	0.657*****	1.000						
	(0.000)	(0.000)	(0.000)	(0.000)							
(V6)	0.292*****	0.287*****	0.301*****	0.518*****	0.665*****	1.000					
	(0.000)	(0.000)	(0.000)	(0.000)	(0.000)						
(V7)	0.243*****	0.245*****	0.238*****	0.359*****	0.375*****	0.366*****	1.000				
	(0.000)	(0.000)	(0.000)	(0.000)	(0.000)	(0.000)					
(V8)	0.253*****	0.247*****	0.249*****	0.382*****	0.392*****	0.372*****	0.523*****	1.000			
	(0.000)	(0.000)	(0.000)	(0.000)	(0.000)	(0.000)	(0.000)				
(V9)	0.256*****	0.192*****	0.235*****	0.244*****	0.228*****	0.190*****	0.157*****	0.171*****	1.000		
	(0.000)	(0.000)	(0.000)	(0.000)	(0.000)	(0.000)	(0.000)	(0.000)			
(V10)	0.259*****	0.213*****	0.231*****	0.250*****	0.231*****	0.186*****	0.176*****	0.176*****	0.698*****	1.000	
	(0.000)	(0.000)	(0.000)	(0.000)	(0.000)	(0.000)	(0.000)	(0.000)	(0.000)		
(V11)	0.176*****	0.139*****	0.181*****	0.133*****	0.156*****	0.149*****	0.078*****	0.136*****	0.358*****	0.317*****	1.000
	(0.000)	(0.000)	(0.000)	(0.000)	(0.000)	(0.000)	(0.000)	(0.000)	(0.000)	(0.000)	

Pairs = 392,654

****p* < 0.01, ***p* < 0.05, **p* < 0.1

**Table 6:** Macro level pairwise correlations of all variables

Variables	(1)	(2)	(3)	(4)	(5)	(6)	(7)	(8)	(9)	(10)	(11)
(V1)	1.000										
(V2)	0.907***	1.000									
	(0.000)										
(V3)	0.935***	0.910***	1.000								
	(0.000)	(0.000)									
(V4)	0.743***	0.682***	0.721***	1.000							
	(0.000)	(0.000)	(0.000)								
(V5)	0.793***	0.749***	0.758***	0.918***	1.000						
	(0.000)	(0.000)	(0.000)	(0.000)							
(V6)	0.757***	0.735***	0.767***	0.801***	0.876***	1.000					
	(0.000)	(0.000)	(0.000)	(0.000)	(0.000)						
(V7)	0.744***	0.682***	0.713***	0.674***	0.696***	0.722***	1.000				
	(0.000)	(0.000)	(0.000)	(0.000)	(0.000)	(0.000)					
(V8)	0.641***	0.586***	0.655***	0.716***	0.720***	0.704***	0.676***	1.000			
	(0.000)	(0.000)	(0.000)	(0.000)	(0.000)	(0.000)	(0.000)				
(V9)	0.595***	0.515***	0.652***	0.463***	0.411***	0.549***	0.441***	0.401***	1.000		
	(0.000)	(0.000)	(0.000)	(0.000)	(0.000)	(0.000)	(0.000)	(0.000)			
(V10)	0.570***	0.539***	0.612***	0.413***	0.384***	0.488***	0.448***	0.320***	0.876***	1.000	
	(0.000)	(0.000)	(0.000)	(0.000)	(0.000)	(0.000)	(0.000)	(0.000)	(0.000)		
(V11)	0.633***	0.654***	0.717***	0.592***	0.601***	0.689***	0.474***	0.653***	0.583***	0.524***	1.000
	(0.000)	(0.000)	(0.000)	(0.000)	(0.000)	(0.000)	(0.000)	(0.000)	(0.000)	(0.000)	

Pairs = 231

******p* < 0.01, ***p* < 0.05, **p* < 0.1

**Table 7:** Confirmatory factor analysis output table

Standardized measurement	OIM					
	Coef	Std.Err	z	*p* > z	[95%Conf	Interval]
(V1)						
L1	0.768	0.001	751.170	0.000	0.766	0.770
	2.072	0.003	714.660	0.000	2.066	2.077
(V2)						
L1	0.681	0.001	596.620	0.000	0.679	0.683
	2.100	0.003	717.760	0.000	2.095	2.106
(V3)						
L1	0.768	0.001	753.030	0.000	0.766	0.770
	2.427	0.003	747.640	0.000	2.421	2.433
(V4)						
L2	0.745	0.001	817.280	0.000	0.743	0.746
	1.714	0.003	667.460	0.000	1.709	1.719
(V5)						
L2	0.844	0.001	1147.530	0.000	0.843	0.846
	1.921	0.003	696.820	0.000	1.915	1.926
(V6)						
L2	0.745	0.001	819.170	0.000	0.743	0.746
	2.302	0.003	737.300	0.000	2.296	2.308
(V7)						
L2	0.510	0.001	369.840	0.000	0.507	0.513
	2.346	0.003	741.070	0.000	2.340	2.352
(V8)						
L2	0.525	0.001	387.530	0.000	0.522	0.528
	2.083	0.003	715.890	0.000	2.077	2.089
(V9)						
L3	0.854	0.001	668.620	0.000	0.851	0.856
	2.126	0.003	720.480	0.000	2.120	2.132
(V10)						
L3	0.814	0.001	644.140	0.000	0.812	0.817
	2.094	0.003	717.100	0.000	2.088	2.100
(V11)						
L3	0.300	0.002	178.940	0.000	0.297	0.304
_cons	3.122	0.004	777.050	0.000	3.114	3.130
var(V1)	0.410		0.002	0.406	0.413	
var(V2)	0.536		0.002	0.533	0.539	
var(V3)	0.410		0.002	0.407	0.414	
var(V4)	0.446		0.001	0.443	0.448	
var(V5)	0.287		0.001	0.285	0.289	
var(V6)	0.445		0.001	0.443	0.448	
var(V7)	0.740		0.001	0.737	0.743	
var(V8)	0.724		0.001	0.722	0.727	
var(V9)	0.271		0.002	0.267	0.275	
var(V10)	0.337		0.002	0.333	0.341	
var(V11)	0.910		0.001	0.908	0.912	
var(L1)	1		.	.	.	
var(L2)	1		.	.	.	
var(L3)	1		.	.	.	
cov(L1,L2)	0.544	0.002	352.960	0.000	0.541	0.547
cov(L1,L3)	0.391	0.002	217.680	0.000	0.388	0.395
cov(L2,L3)	0.356	0.002	202.430	0.000	0.353	0.360

LR test of model vs. saturated: chi^2^(41) = 81,486.17, Prob > chi^2^ = 0.0000

Multilevel regression model

$${SCI}_{itk}= {\beta }_{0k}+ {\beta }_{1tk} + {e}_{itk}$$

$${\beta }_{0k} {=\gamma }_{ 00}+ {\mu }_{0k}$$

$${\beta }_{1tk} {=}{\gamma }_{10} {gdp}_{tk} +{\mu }_{1tk}$$

where ${SCI}_{itk}$ is the outcome of an individual $i$ within a country-wave $t$ within a country $k$; ${\beta }_{0k}$ is the intercept for country $k$; ${\beta }_{1tk}$ is decomposable into the random effect for country-wave ${\mu }_{1tk}$, and ${\gamma }_{10}$, the coefficient of the control variable (GDP p.c.). The country intercept ${\beta }_{0k}$, is decomposable into the overall intercept ${\gamma }_{ 00}$ and a random effect for country ${\mu }_{0k}$ (Tables 8, 9, 10).

**Table 8:** Summary statistics for all variables used in the regression analysis

Variable	Obs	Mean	Std. Dev	Min	Max
*Micro*					
SCI (micro)	371,888	.525	.158	0	1
Social Trust (micro)	371,888	.523	.181	0	1
Institutional Trust (micro)	371,888	.524	.194	0	1
Openness (micro)	371,888	.528	.205	0	1
Age (micro)	432,490	48.174	18.609	13	123
Gender (micro)	434,107	.538	.499	0	1
Education (years)	429,575	12.344	4.059	0	60
Income (difficulty)	425,788	2.079	.888	1	4
Unemployed (micro)	434,393	.022	.146	0	1
Discriminated (micro)	434,393	.071	.257	0	1
Subjective wellbeing (micro)	429,831	.703	.2	0	1
*Macro*					
GDP p.c. (control)	433,110	34.305	12.457	6.434	73.297
Post-communist	434,346	.278	.448	0	1
Gini Coefficient	424,104	29.706	3.877	23	42.1
Gender Inequality Index	386,661	20.538	15.094	1	93
Welfare expenditure	338,403	1483.57	1070.177	48.682	4797.054
SCI (macro)	434,346	.52	.081	.34	.687
Social Trust (macro)	434,346	.518	.085	.322	.694
Institutional Trust (macro)	434,346	.517	.103	.232	.729
Openness (macro)	434,346	.524	.079	.319	.719

**Table 9:** Pairwise correlation and lagged multivariate linear regression of income inequalities and entropy of cohesion orientations by country-wave observations

Pairwise correlations		
Variables	(1)	(2)
(1) Gini (solt)	1.000	
(2) SCI (entropy)	0.458**	1.000

Multiple Regression						
SCI (entropy)	Coef	St.Err	*t*-value	*p*-value	[95% Conf	Interval]
L. Gini (solt)	.001**	.001	2.22	.028	000	.002
L.GDP p.c	− .001***	000	− 9.10	000	− .002	− .001
L. Education index	− .000***	000	− 2.91	.004	− .001	0
Constant	.224***	.029	7.83	000	.168	.281
Mean dependent var	0.145		SD dependent var		0.033	
R-squared	0.525		Number of obs		177	
F-test	63.805		Prob > F		0.000	
Akaike crit. (AIC)	− 835.348		Bayesian crit. (BIC)		− 822.644	

****p* < .01, ***p* < .05, **p* < .1

**Table 10:** Summary statistics for regional variation (coefficient of variation) of SCI and its components by country

Country	SCI (macro)	CV (SCI)	CV (SOC.T)	CV (INS.T)	CV (OPEN)	SCIva	Difference (SCIva–SCI)
Albania	0.464	0.092	0.115	0.164	0.053	0.421	− 0.043
Austria	0.572	0.038	0.033	0.059	0.091	0.550	− 0.022
Belgium	0.559	0.055	0.091	0.054	0.055	0.528	− 0.031
Bulgaria	0.393	0.182	0.188	0.206	0.258	0.321	− 0.072
Switzerland	0.645	0.025	0.023	0.051	0.101	0.629	− 0.016
Czech Republic	0.480	0.073	0.076	0.107	0.069	0.445	− 0.035
Germany	0.573	0.067	0.047	0.071	0.099	0.534	− 0.038
Denmark	0.659	0.034	0.021	0.027	0.068	0.637	− 0.022
Estonia	0.539	0.064	0.056	0.062	0.083	0.504	− 0.035
Spain	0.539	0.109	0.120	0.159	0.090	0.481	− 0.059
Finland	0.673	0.035	0.034	0.040	0.049	0.649	− 0.024
France	0.527	0.056	0.047	0.058	0.077	0.498	− 0.029
United Kingdom	0.566	0.045	0.040	0.045	0.076	0.541	− 0.026
Greece	0.338	0.167	0.194	0.194	0.204	0.282	− 0.057
Hungary	0.463	0.143	0.152	0.176	0.168	0.396	− 0.066
Ireland	0.578	0.044	0.035	0.029	0.085	0.553	− 0.025
Italy	0.491	0.069	0.061	0.064	0.085	0.457	− 0.034
Lithuania	0.491	0.032	0.067	0.076	0.124	0.475	− 0.016
Netherlands	0.632	0.021	0.023	0.024	0.029	0.618	− 0.013
Norway	0.677	0.023	0.022	0.027	0.036	0.661	− 0.016
Poland	0.491	0.064	0.104	0.091	0.093	0.459	− 0.032
Portugal	0.520	0.051	0.043	0.052	0.064	0.494	− 0.026
Serbia	0.419	0.079	0.116	0.011	0.115	0.385	− 0.033
Russia	0.421	0.048	0.059	0.072	0.110	0.400	− 0.020
Sweden	0.657	0.019	0.020	0.021	0.031	0.644	− 0.012
Slovenia	0.457	0.069	0.053	0.059	0.136	0.426	− 0.032
Slovakia	0.410	0.062	0.145	0.079	0.065	0.384	− 0.026
Ukraine	0.367	0.123	0.193	0.200	0.116	0.322	− 0.045
Kosovo	0.397	0.185	0.139	0.206	0.237	0.324	− 0.073

Coefficient of variation

$${{SCI}_{sd}= \sqrt{\frac{\sum_{ i=1}^{ n}{\left({ x}_{i}-\bar{x}\right)}^{2}}{n-1}} SCI}_{cv}= \frac{{SCI}_{sd}}{{SCI}_{\upmu }}$$

where ${SCI}_{sd}$ is the standard deviations in the cohesion orientations for the subpopulations (groups, regions, languages, classes) in a society, and ${SCI}_{\upmu }$ is the mean of cohesion orientations in a given society (Figs. 7, 8, 9, 10, 11).
